# AGE DIFFERENCES IN PRO-ENVIRONMENTAL BEHAVIORS: IS IT ABOUT ME OR THE NEXT GENERATION?

**DOI:** 10.1093/geroni/igae098.4373

**Published:** 2024-12-31

**Authors:** Zhixuan Lin, Helene Hoi-Lam Fung

**Affiliations:** University of Zurich, Zurich, Zurich, Switzerland; The Chinese University of Hong Kong, Hong Kong, China (People’s Republic)

## Abstract

Aging society and climate change have emerged as two pressing concerns in modern society. However, questions remain unsolved regarding whether older and younger adults engage in pro-environmental behavior with different frequencies and/or out of different motivations. In the current study, we examined age differences in pro-environmental behaviors, as well as two underlying motivations: self-relevance and self-transcendence motivations. The former is defined as the extent to which people feel personally harmed or worried by climate change (i.e., ecological risk perceptions), and the latter is defined as the extent to which people hold concerns about the next generation. According to the positivity effect in the aging process and the socioemotional selectivity theory, older age could be correlated with lower risk perceptions and higher generativity concerns. These factors indicate opposing directions in age-related differences in pro-environmental behaviors. We conducted a survey among 126 older adults (Mean age = 70.65, SD = 5.12, 61.5% females) and 117 younger adults (Mean age = 25.43, SD = 5.93, 67.2% females). The results revealed that older adults engaged in pro-environmental behaviors more frequently and scored higher on social generativity concerns compared to younger adults. However, no significant age difference was found in ecological risk perceptions. Moreover, social generativity concerns positively correlated with pro-environmental behaviors only among younger adults, but not among older adults. Ecological risk perceptions positively correlated with pro-environmental behaviors among both younger and older adults. These results provide insights into different approaches for encouraging pro-environmental behaviors among younger and older adults.

